# Evaluating the Impact of Training Teachers to Identify Learning Disabilities: A Pre-experimental Study on Knowledge Enhancement

**DOI:** 10.7759/cureus.55685

**Published:** 2024-03-06

**Authors:** Akanksha P Dani, Yamini v Pusdekar, Ketan R Dagdiya, Vishwajit R Deshmukh

**Affiliations:** 1 Community Medicine, All India Institute of Medical Sciences, Nagpur, IND; 2 Community Medicine, Narendra Kumar Prasadrao (NKP) Salve Institute of Medical Sciences and Research Centre, Nagpur, IND; 3 Anatomy, All India Institute of Medical Sciences, Nagpur, IND

**Keywords:** knowledge, early identification, quasi-experimental study, referral, screening, pre-post, school teachers, learning disability

## Abstract

Introduction

Learning disability (LD) affects many school-going children and is seldom recognized or treated. As teachers spend time with students, they can easily recognize LD by observing academic activities and behaviors. In this context, the present study was conducted to assess the knowledge and practices of teachers regarding LD and evaluate the impact of an educational intervention on teachers' knowledge regarding LD and its screening and referral.

Methods

A pre-experimental study, including pre-post interventional assessments of teachers, was conducted from June 2018 to December 2019. A universal sample of 150 teachers from 10 schools teaching primary (first to fifth grade) and upper primary (sixth to eight grade) grades was included. Their knowledge about LD was assessed using the Dyslexia Assessment for the Languages of India (DALI), and an educational intervention for assessing, screening, and identifying LD was implemented. Data was analyzed using SPSS version 24.0 (IBM Inc., Armonk, New York). Using descriptive statistics (mean, median, and standard deviation). The pre-post test results were compared using the McNemar test.

Results

Overall knowledge about LD was 24.7% at baseline, and improved to 76% post-intervention (p<0.001). The knowledge for most of the components showed improvement. Teachers with a good level of knowledge increased from 21% to 84%. Post-intervention screening of students increased from 0.53% to 13.37%. The suspicion rate for LD increased from 0.04% to 1.94% post-intervention.

Conclusion

Knowledge about LD was poor among the school teachers. However, the overall knowledge about LD, its specific domains, screening as well as actual LD screening significantly improved after the intervention (p<0.001). This emphasizes the need of training primary and post-primary school teachers about LD and the services available for children with LD.

## Introduction

Disability constitutes an intricate and multifaceted construct. Individuals with disabilities confront pervasive discrimination driven by societal preconceptions and biases. The sphere most susceptible to such discrimination is the realm of 'education' [1]. Specific learning disability (SPLD) or learning disability (LD), used interchangeably, comprises a cluster of neurodevelopmental conditions that emerge during childhood, characterized by persistent difficulties in acquiring efficient reading (dyslexia), writing (dysgraphia), or mathematical calculation skills (dyscalculia). These challenges persist despite normal cognitive abilities, conventional education, intact sensory faculties, adequate motivation, and sociocultural opportunities [2]. In India, 2%-18% of children experience these issues, yet only a minority undergo proper screening and detection [3].

These children often remain unidentified, their struggles manifesting as 'academic problems' such as slow and inaccurate reading, line-skipping while reading aloud, frequent spelling errors, illegible handwriting with poor sequencing, and an inability to perform even basic arithmetic operations [4]. Children with SPLD consistently underachieve in their academic pursuits relative to their intellectual potential. Their 'academic problems' also negatively affect their quality of life, including self-esteem, peer and family relationships, and social interactions [5]. Teachers spend the majority of their day with students and are ideally positioned to observe academic activities and behaviors. They are the first to notice changes and can compare students with their peers. Training teachers to address the requirements of children with learning disabilities is the optimal approach for identifying and assisting these children.

The Rights of Persons with Disabilities Act (RPWD) of 2016 and the Right to Education (RTE) Act of 2009 stipulate that all children with disabilities should be identified at the earliest opportunity, and their specific needs arising from disabilities should be appropriately addressed to support them in realizing their full potential [6]. Early identification of students with LD should occur during primary grades, but a definitive diagnosis of specific LD should be deferred until the child reaches the third grade, around seven to eight years old, as some children are "normal late developers" who naturally overcome their learning difficulties (unlike SPLD, which is a lifelong condition) [6-9]. Therefore, teachers of all grade levels should possess the requisite knowledge to screen students in their classes.

In the sphere of academic discourse, it is also recognized that there exists a spectrum of learning variations among students, stemming from the distinctive cognitive profiles inherent to each individual. These variations manifest diversely, encompassing preferred modalities of learning, ranging from auditory to visual inclinations, and may also be influenced by extracurricular interests alongside disparities in neurological development. Notably, students exhibiting such divergent learning proclivities often demonstrate remarkable intellectual acumen and innovative potential [10].

Comprehending this array of learning diversities is imperative for educators in devising an inclusive pedagogical framework that accommodates the heterogeneous learning needs of their students [11-13]. Empirical evidence suggests that educational outcomes are optimized when instructional methodologies align with students' preferred learning modalities, as opposed to instances of incongruence. Hence, educators must adeptly discern between instances of genuine learning disabilities and inherent learning variations to effectively facilitate their students' academic progress.

Literature has shown multiple cross-sectional studies throughout India that highlight the occurrence of learning disabilities among school children in different states. However, only a limited number of studies have focused on interventions to address this issue. In this context, the present study was designed to assess the understanding of school teachers regarding screening of LD and its referral and to study the effectiveness of an educational intervention on teachers' knowledge regarding screening and referral for LD. 

## Materials and methods

Study design

The research employs a pre-experimental study design, encompassing pre and post-interventional assessments of teachers devoid of a control group.

Duration of study

The study encompassed a period of 18 months from June 2018 to December 2019, during which many activities were undertaken, including compilation of background data, preparation of the research protocol, obtaining approval from an Institutional Ethics Committee, data collection, implementation of interventions, data collection after intervention and analysis of the collected data.

Sampling technique

Using multistage sampling from all the wards under the Brihan Mumbai corporation, five wards were randomly selected in the first stage. A list of all the schools under BMC in the selected wards was taken from the education office. Then, ten schools, including two randomly selected schools from each of the shortlisted wards, were selected using a random number table. 

Study population

The study focused on teachers in Municipal Schools, specifically targeting primary (first to fifth grade) and upper primary (sixth to eighth grade) teachers. All teachers consent to participate in the study and are ready to follow up for six months.

Inclusion and exclusion criteria

All the teachers from the selected schools teaching students from first to eighth grades who consented to the study were included. Teachers on long leave and those who could not be contacted during the study period were excluded from the study.

Sample size and ethical statement

The study was conducted after obtaining ethical clearance from the Institutional Ethics Committee (Registration no. ECR/417/INST./MH/2013), outward no - IEC (II/OUT/303/18). Permission for conducting the study was sought from each school's principal and the education officer. A universal sample of all the school teachers from the selected schools was considered for the study. All teachers from these schools were contacted, and the study's purpose was explained to them. After obtaining written informed consent, all 167 teachers were requested to fill out a predesigned and pretested questionnaire. Out of the total questionnaires, 17 were incomplete and hence were not considered for analysis. So, the adequate sample size was 150.

Study procedure

Interaction with the principals of the teachers' training program was done to get insights on the inclusion of learning disabilities in the syllabus. A visit to the Learning Disability clinic run by Seth G.S. Medical College and KEM Hospital, Mumbai, India, was made, and the procedure for diagnosis and certification was understood by interacting with the pediatricians, psychologists, occupational therapists, and special educators involved to get the depth of the topic. Also, training for the administration and use of the Dyslexia Assessment for the Languages of India (DALI) screening tool has been taken, and official permission to use the tool in the present study has been obtained. The semi-structured questionnaire was designed based on a literature review and the DALI screening tool [14,15]. The questionnaire for teachers consists of questions related to knowledge of teachers regarding the concept of LD, symptoms, referral, provisions, criteria used to detect, remedial measures, training, etc. It was developed based on the DALI screening tool along with the information on the sociodemographic variables and was pretested for simplification of language and for ensuring its understanding by the teachers. The English version of the questionnaire was also translated into Hindi and Marathi for ease of administration to the teachers. 

The intervention was planned during the first interaction with teachers, and it comprises comprehensive sessions on LD consisting of signs and symptoms for identification, screening tools, referral, management, and remedial education. The session follows the following sequence:

General introduction--> introduction to LD--> consequences of LD--> need for teachers --> awareness about symptoms of LD--> how to suspect students with probable LD using the DALI screening tool--> management and provisions for students with LD --> teachers' remarks and open discussion regarding practical problems. 

**Figure 1:**
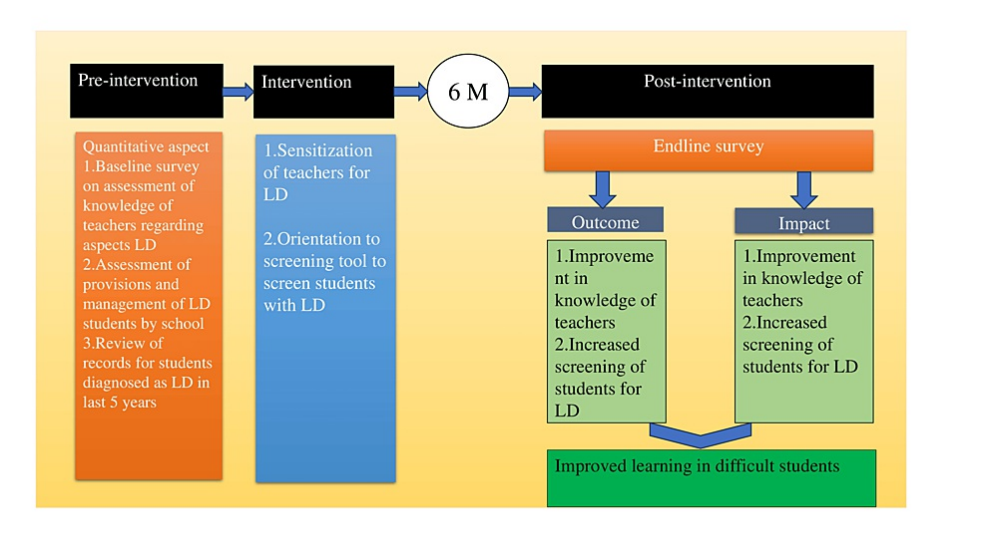
Theory of change of knowledge of the teachers after intervention LD - learning disability

Expected outcomes were improvement in the comprehensive knowledge of teachers regarding LD and improvement in the rate of screening and, thus, timely detection and referral of LD among needy students. A theory of change was conceptualized to improve teachers' knowledge of detecting LD among the assessed children (Figure 1). After assessing initial knowledge and implementing the intervention, teachers should be able to suspect and screen the students with learning difficulties better than they have screened in the last five years.

Thus, in the first visit, teachers' knowledge was pretested with a semi-structured questionnaire, then intervention was given, and after six months, the same teachers were reviewed for their knowledge. 

Operational definitions

Level of Overall Knowledge

The overall knowledge level was decided using the scoring for the domains such as defining learning disability, symptoms, detection criteria, referral processes, provisions, and remedial measures. Considering all the above domains, teachers with scores of more than 50% were considered to have good knowledge, and those with lower scores were considered poor. Children with suspected LD were decided based on the scoring of the DALI scale. 

Scoring Using the DALI Scale

DALI stands for Dyslexia Assessment in the Languages of India. The National Brain Research Centre (NBRC), New Delhi, developed it. There are two screening tools: 

1. The Junior Screening Tool (JST) for the screening of LD between five to seven-year-old children.

2. The Middle Screening Tool (MST) for screening LD in upper grades.

It has been developed and validated to identify children with LD. It has been standardized and validated across the four languages - Hindi, Marathi, Kannada, and English. It is in the form of scale scoring 0, 1, and 2. The cut-off for JST is 12, and MST is 23. The child who scores more than the cut-off score for that school may have LD and need to be referred for diagnostic assessment. The teacher must have observed the students for at least three months before filling out the tool. The tool was filled out based on the teacher's observation about the child's behavior, comparing the child's performance with his fellow batchmates and considering the child's background (socio-economic status, the environment at home, etc.) [14,15]. 

Statistical analysis

Data was analyzed using SPSS version 24.0 (IBM Inc., Armonk, New York). Variables were coded and then analyzed using descriptive statistics such as mean, median, and standard deviation. Pre and post-test results were compared using the McNemar test.

## Results

The study was conducted among 150 school teachers from 10 randomly selected schools from the study area. In the present study, the assessment of the knowledge of teachers was done at the first interaction with the teachers. Then, after intervention, teachers were asked to observe the students for at least four to five months and to fill out the DALI screening tool for students showing the signs and symptoms of LD. Observing students for a longer duration will help teachers make the correct decision. After six months, teachers' knowledge was assessed, and teachers were asked for the number of students for whom they suspected LD and filled out the DALI screening tool.

Teachers in the selected schools were almost equally distributed as per age (Table 1). The mean age of teachers was 41.8 years, with a standard deviation of 9.878 years. The teachers' ages ranged from 25 to 58. There was a female preponderance among the school teachers. Most teachers belonged to Urdu medium schools as the study area has a significant population of the same religion. All the teachers were qualified and experienced, with 39.3% of teachers having a bachelor's degree in education and nearly 58%(n=87) holding a diploma in education. The mean years of experience of the teachers was 17 years. Experience-wise, teachers with mixed levels of experience, i.e., young teachers with only one to 10 years of experience and those with experience of > 20 years, were also present in the selected schools.

**Table 1:** Sociodemographic parameters of teachers (N=150)

Characteristics	Number of teachers (N=150)	Percentage
Age		
<40 years	67	44.7%
>40 years	83	55.3%
Age (mean + SD): 41.8 + 9.878; range 25-58 years		
Sex		
Male	60	40%
Female	90	60%
Language of school		
Urdu	77	51%
Hindi	32	21%
English	23	15%
Marathi	18	12%
Educational qualification		
Diploma in education	87	58%
Bachelors in education	59	39.3%
Masters in education	4	2.7%
Class of teaching		
1^st^-5^th^ class	90	60%
6^th^-8^th^ class	60	40%
Teaching experience		
1-10 years	53	35.3%
10-20 years	35	23.3%
20-30 years	55	36.7%
>30 years	7	4.7%
Years of teaching (mean + SD): 17 +10 years		

It was observed that knowledge regarding the components of the definition of LD has significantly increased for almost all the domains, such as reading, writing, mathematics, comprehension, and memorizing, except for speaking and pronunciation. An analysis of teachers' responses regarding individual elements contributing to learning disabilities (LD) reveals that among six potential components (reading (R), writing (W), mathematics (M), pronunciation (P), comprehension (C), and memorizing (Mz)), during pre-intervention phase 40 teachers singled out just one component as the primary determinant of LD (Table 2). A smaller number of teachers considered combinations of two, three, or four of the abovementioned factors as the determinants of LD. Most of them emphasized reading, writing, and mathematical problem-solving difficulties. Pronunciation, memorizing, and comprehension were also mentioned, but to a significantly lesser extent. Notably, none of the teachers identified all six components contributing to learning disability during pre-intervention. After the intervention, there was a significant increase in several teachers who recognized the greater number of components correctly. In the pre-intervention phase, only three teachers identified four components, but in the post-intervention phase, 45 teachers could identify four or more four components correctly.

**Table 2:** Knowledge of school teachers with respect to different domains on the definition of LD (n=150) p<0.05 is considered significant and p<0.001 is considered highly significant with the McNemar test

Characteristics	Pre-intervention		Post-intervention		p-value
Difficulty in reading	37	24.67%	93	62%	p<0.001
Difficulty in writing	40	26.67%	102	68%	p<0.001
Difficulty in mathematics	10	6.67%	62	41.3%	p<0.001
Difficulty in comprehension	34	22.66%	70	46.7%	p<0.001
Difficulty in speaking/pronunciation	15	10%	15	10%	p>0.05
Difficulty in memorizing	34	22.66%	44	29.3%	p<0.001
Overall	37	24.7%	114	76%	p<0.001

**Figure 2:**
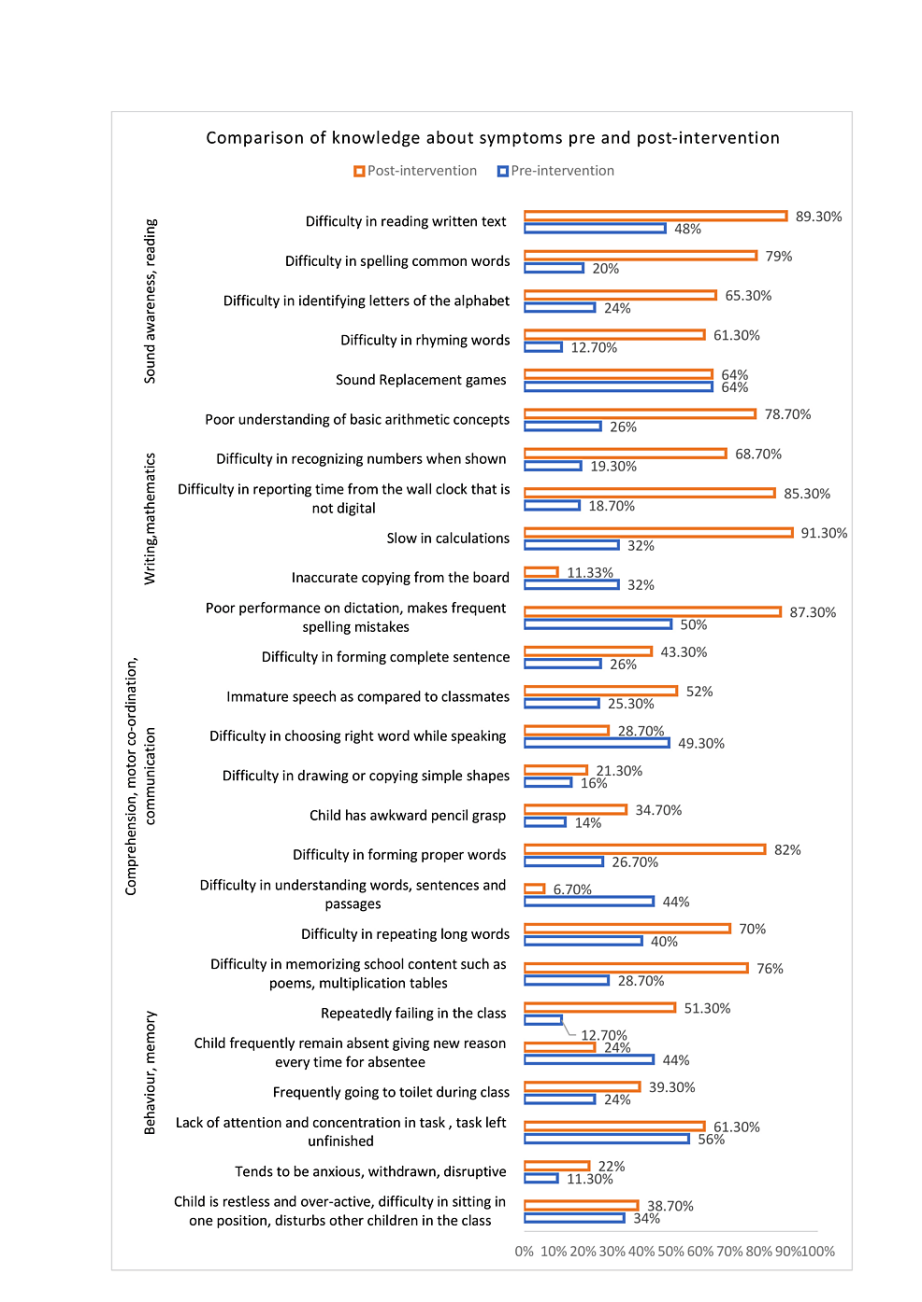
Comparing the change in knowledge regarding symptoms of LD among teachers LD - learning disability

In comparing knowledge regarding symptoms, questions were asked to the teachers, and they needed to identify if this type of behavior belonged to the category of symptoms indicating LD or not. It was observed that there was a significant increase in knowledge regarding symptoms of comprehension, motor co-ordination, communication, writing, mathematics, reading, and sound awareness; there is a noticeable increase in post-intervention knowledge except for a few symptoms such as difficulty in choosing the right words, difficulty in drawing or copying simple words or figures, and difficulty in understanding words, sentences, and passages (Figure 2).

On comparing the teachers with good knowledge in the pre-and post-intervention phases, most of the component knowledge was improved. Teachers with good overall knowledge regarding LD increased from 21% (n=32) to 84% (n=127) (Table 3).

**Table 3:** Comparison of overall the level of knowledge of school teachers considering comprehensive components (n=150) p<0.05 is considered significant by the McNemar test LD - learning disability

Characteristics	Teachers with good knowledge			
	Pre-intervention		Post-intervention	
Components of the definition of LD	37	24.7%	114	76%
Symptoms	31	11.3%	141	94%
Criteria used for detection	31	20.7%	80	54.34%
Referral	18	12%	57	38%
Provisions	11	7.3%	88	58.67%
Remedial measures	61	40.7%	74	49.34%
Overall	32	21.4%	127	84.67%

**Figure 3:**
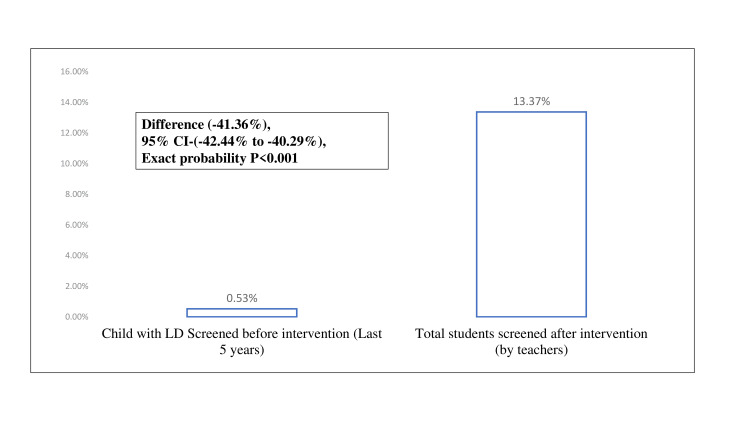
Comparison of students screened for LD in the last five years to the students screened post-intervention LD - learning disability

**Figure 4:**
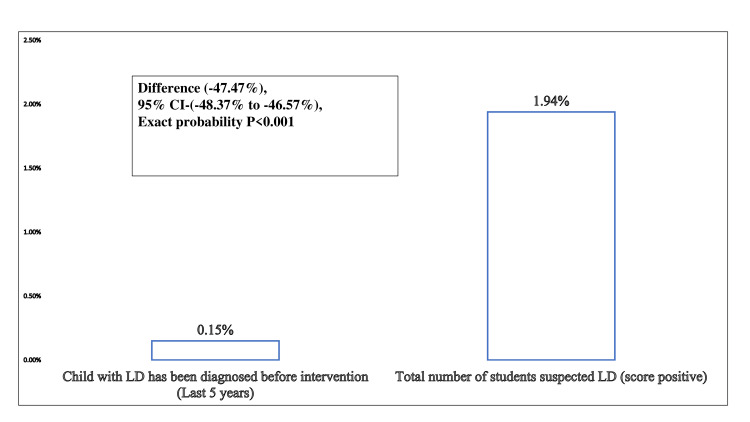
Children detected or suspected in schools by a teacher for LD pre- and post-intervention LD - learning disability

On screening of students before five years and after the intervention, it has increased from 0.53% (n=35) to 13.37% (n=873) (Figure 3). The suspicion rate increased from 0.15% (n=10) to 1.94% (n=127) after using a specific screening tool (Figure 4). After the intervention, there was a significant overall improvement in teachers' knowledge and its application in terms of improved screening and referrals.

Thus, the proposed change in knowledge and its application in increasing knowledge, screening, and improved learning by giving appropriate referrals and provisions to needy students has created a more inclusive and effective educational environment, ultimately enhancing the overall academic experience for all students.

## Discussion

The study was conducted to assess the knowledge about LD among 150 teachers working in primary and upper primary schools from the randomly selected schools in the study area. An educational intervention comprising of comprehensive sessions on LD consisting of signs and symptoms for early identification of LD, screening tools, referral procedures, and places for referral, management, and remedial education for LD were conducted for the teachers of selected school. The impact of this intervention on teachers' knowledge regarding LD, the rate of screening for LD, and making referrals for LD after the intervention was assessed. 

The sociodemographic characteristics of the study participants revealed a mean age of 41.8 years with a standard deviation of 9.87 years with a female preponderance. Most of the teachers belonged to Urdu medium schools as the study area has a major population of the same religion. All the teachers were qualified and experienced, with more than half of them having a bachelor's degree in education and nearly 37% holding a diploma in education. The mean years of experience of the teachers was 17 years (Table 1). These characteristics were similar to the study conducted by Koshy et al. in 2021 among primary school teachers from public schools, which revealed the age nearly 54.0% of the teachers to be between 31-50 years, 59.33% of the teachers were females, 0.66% of teachers were graduate, 20% teachers had done a diploma, and 31.33% were post- graduates [16]. Experience-wise, the majority of them (72.66%) had experience of more than five years, while 20.66% had experience of three to five years. Except for the experience, other factors were similar to the present study. Another study by Madhamani et al. also depicted similar sociodemographic except for a male preponderance [17]. The sociodemographic characteristics showed striking similarity with other recent studies conducted by Dhindsa et al. (2022) and Rosemary et al. (2021) [18,19]. Experienced teachers were able to identify the altered behavior and academic performance of the students more effectively with their engagement with students. But, the majority of the teachers were young and, therefore, there was a need for training the teachers in the identification of LD and referring these children to the appropriate center for further management [18,19]. This reinforces the need for in-service training of the school teachers to identify children with LD and refer them to appropriate centers for further management. These trained teachers can also be utilized to provide effective interventions at the school itself for the affected children and will prove useful to circumvent the need for additional resources in the already burdened and resource-poor health care settings. 

At baseline, only 24.7% of the teachers were aware of the different components of LD. In the study by Madhamani et al. 33. 5% of the teachers had adequate knowledge, whereas it was moderate among 45.0% of them [17]. Also, knowledge regarding the components of the definition of LD had significantly increased for almost all the domains, such as reading, writing, mathematics, comprehension, and memorizing, except for speaking and pronunciation after the intervention, with an overall improvement from 24.7% to 76.0% (p<0.001). An increase in knowledge about the various components of definition will help teachers suspect the students with learning difficulties. Trained teachers will be useful for engaging them in screening these LDs and identifying the children at earlier stages for instituting effective interventions. 

A comparison of teachers with good knowledge in pre- and post-intervention phases reveals that for most of the components, knowledge was improved. Teachers with good levels of overall knowledge regarding LD were increased from 21% to 84%.

The results of the study by Bhavya et al. showed that 64% of teachers had an average level of knowledge, which was consistent with the present study in which nearly 49% of teachers had average scores [20]. Also, in other studies done in South India, there is a substantial increase in knowledge and attitudes of teachers towards learning disability from 35 to 83%, which is consistent with the findings of the present study [21,22]. However, most of the studies are done in private school settings where there are ample resources for teachers and students are available to screen and manage LD. While very few studies are done in government or municipal settings where there are constraints of material, economic, and human resources [23]. 

Barriers to the less knowledge regarding LD definitions, and symptomatology reported by teachers were strength of class, repeated meetings for various trainings, very few refresher trainings on LD that too for a limited number of teachers, lack of in-house counsellors and lack of structured criteria for screening and referral. 

In the present study, screening of students before five years and after the intervention has been increased from 0.53% to 13.37%. Also, the suspicion rate has increased from 0.15% to 1.94% after the use of specific screening tools. Most of the quasi-experimental studies have measured outcomes in terms of knowledge, attitude, and practices but not in terms where children were suspected by teachers and were referred through proper mechanisms [1-3]. The intervention has channelized the enhancement of the knowledge of teachers and existing mechanisms for referral and management of LD children [24,25]. Even after the screening and referral of a child, the challenges of the teachers were counseling the parents and convincing them to get help [26-28]. The teachers expressed the readiness to learn about the recognition of LD among their pupils and were eager to undergo training for the same [29,30]. This shows the positive attitude of the teachers and their will to help the children with LD overcome their shortcomings. Therefore, organizing trainings and refresher trainings for the identification of LD and dissemination of knowledge regarding the services available for children with LD would empower the school teachers to identify and refer children with LD. At the same time, these teachers would serve as a tangible workforce for screening and management of LD as they can also be trained in implementing improvement programs for children with LD. 

Strengths and limitations of the study

The study highlights the importance of refresher trainings for teachers for early detection of learning disability among children. The study was conducted only in public schools, and the findings are therefore not generalizable to private schools.

Implications

The study emphasized the potential role of teachers as change makers by detecting and referring children with LD at appropriate centers to limit their disability and provide them an opportunity for inclusive education. Also, the distinction between learning variations and actual learning disability is crucial. However, the teachers can only be trained to screen the children, and the suspected children need further expert evaluation for management. Therefore, empowering teachers to screen children will yield suspected children who need further attention for assessment of learning disability, thereby optimizing the existing resources and utilizing them appropriately. At the same time, it prevents the burden on the experts of screening school children. Futuristic steps can be taken to develop and validate curricula for teachers, focusing on the distinction of learning variations and disability.

Future directions

A follow-up study for assessing the outcomes in students with suspected LD can be planned. Another area that needs attention is the study of barriers at a familial level for the diagnosis and management of LD to facilitate parental involvement in the same.

## Conclusions

The education system has established protocols for identifying and diagnosing children with learning disabilities (LD), yet a deficiency in awareness and understanding hampers their effective utilization, thereby impeding the identification of vulnerable children. Regular refresher training sessions for educators hold promise in fostering sustained awareness and competency among teachers, thus facilitating prompt and adequate support for students encountering learning challenges. Empowering teachers through comprehensive training enables them to serve as adept resources for both screening and implementing interventions to assist children affected by learning disabilities. This approach not only enhances the likelihood of timely identification and support but also underscores the pivotal role educators play in addressing the needs of students with learning difficulties. Thus, prioritizing teacher training emerges as a fundamental strategy in promoting early intervention and support for children with learning disabilities.
